# Supplementary material for: HIP1R Expression and Its Association with PD-1 Pathway Blockade Response in Refractory Advanced NonSmall Cell Lung Cancer: A Gene Set Enrichment Analysis
Source: J Clin Med. 2020 May 11;9(5):1425. doi: 10.3390/jcm9051425 (PMC7291156; doi:10.3390/jcm9051425)
Supplement: Supplementary file 1 [file jcm-09-01425-s001.pdf]

Supplementary material

Supplementary Table 1. Gene sets within the top 20-ranked list related to low HIP1R in adenocarcinoma patients

NAME	SIZE	ES	NES	Nominal p-val	FDR q-val	FWER p-val
HALLMARK_EPITHELIAL_MESENCHYMAL_TRANSITION	160	0.631063	1.976234	0.005941	0.030414	0.018
**HALLMARK_ALLOGRAFT_REJECTION**	**167**	**0.622762**	**1.95983**	**0.005825**	**0.020678**	**0.027**
HALLMARK_PROTEIN_SECRETION	75	0.539298	1.921606	0.007952	0.021764	0.039
**HALLMARK_INFLAMMATORY_RESPONSE**	**172**	**0.537692**	**1.873854**	**0.013861**	**0.031232**	**0.059**
**HALLMARK_IL6_JAK_STAT3_SIGNALING**	**73**	**0.579944**	**1.827955**	**0.011928**	**0.041183**	**0.1**
HALLMARK_COMPLEMENT	170	0.458096	1.776712	0.009823	0.054888	0.149
HALLMARK_APOPTOSIS	136	0.411506	1.710965	0.005917	0.083721	0.234
HALLMARK_MTORC1_SIGNALING	165	0.493518	1.697493	0.026052	0.081602	0.26
HALLMARK_INTERFERON_GAMMA_RESPONSE	166	0.559316	1.675848	0.063492	0.082374	0.281
HALLMARK_KRAS_SIGNALING_UP	163	0.415849	1.652891	0.017308	0.086092	0.313
HALLMARK_HYPOXIA	159	0.407715	1.627792	0.023346	0.093919	0.354
HALLMARK_ANDROGEN_RESPONSE	71	0.4141	1.599152	0.02549	0.102871	0.408
**HALLMARK_IL2_STAT5_SIGNALING**	**166**	**0.38843**	**1.595789**	**0.034091**	**0.097635**	**0.415**
HALLMARK_MYC_TARGETS_V1	163	0.56081	1.577324	0.073852	0.101525	0.446
HALLMARK_TNFA_SIGNALING_VIA_NFKB	165	0.448866	1.480853	0.118236	0.163775	0.614
HALLMARK_UV_RESPONSE_DN	106	0.370467	1.396578	0.113725	0.239207	0.744
HALLMARK_PI3K_AKT_MTOR_SIGNALING	81	0.335862	1.394414	0.070588	0.227701	0.746
HALLMARK_ANGIOGENESIS	31	0.44204	1.341427	0.151639	0.270267	0.819
HALLMARK_TGF_BETA_SIGNALING	43	0.371833	1.275573	0.178138	0.338608	0.899
HALLMARK_INTERFERON_ALPHA_RESPONSE	76	0.470194	1.244008	0.28937	0.362696	0.924

Abbreviations: ES, enrichment score; FDR, false discovery rate; family-wise error rate, FWER; NES, normalized enrichment score

Supplementary Table 2. Core enrichment gene list of HALLMARK_ALLOGRAFT_REJECTION

Probe	Rank in gene list	Rank metric score	Running es
NCK1	9	0.359524	0.016133
IFNGR1	17	0.346692	0.031773
FCGR2B	28	0.340233	0.046968
CD86	37	0.332375	0.061899
FAS	65	0.315861	0.075134
HLA-DRA	102	0.301541	0.087266
GPR65	118	0.296194	0.100184
ABCE1	236	0.271404	0.106943
RPL39	257	0.267664	0.1183
RPS3A	265	0.266653	0.13025
CCL7	280	0.263992	0.141733
CCL2	289	0.262089	0.153423
IL10	297	0.260421	0.165085
DEGS1	309	0.258553	0.176465
B2M	320	0.256749	0.187811
SRGN	328	0.255015	0.199224
SOCS5	354	0.251267	0.209579
CCR1	426	0.243185	0.217299
CCL13	440	0.241379	0.227789
CCL11	518	0.234165	0.234798
ABI1	542	0.231367	0.244334
NPM1	602	0.226177	0.25186
LCP2	619	0.224889	0.261441
IL15	658	0.222156	0.269815
IGSF6	676	0.221386	0.279186
LY86	682	0.221157	0.289136
HLA-DMB	684	0.220915	0.299273
IL1B	724	0.217414	0.307378
INHBA	742	0.216479	0.316523
CD4	759	0.215482	0.325671

PTPRC	825	0.211518	0.332226
JAK2	910	0.205048	0.337548
DARS	918	0.204617	0.346638
CD80	971	0.201888	0.353388
ST8SIA4	998	0.200772	0.361366
ETS1	1023	0.199474	0.369383
TLR3	1039	0.19833	0.377789
F2R	1055	0.197466	0.386155
HDAC9	1057	0.197367	0.395206
TGFB2	1089	0.195453	0.402692
LYN	1131	0.193173	0.409582
BCL10	1197	0.190418	0.415164
IL2RA	1241	0.187416	0.42169
CRTAM	1299	0.1842	0.427379
CAPG	1414	0.178588	0.430006
CD28	1518	0.173245	0.432927
KLRD1	1523	0.173102	0.440712
CD74	1532	0.17269	0.44828
GBP2	1537	0.17224	0.456025
MRPL3	1547	0.171929	0.463509
GLMN	1557	0.171469	0.470972
EIF3J	1632	0.168421	0.475097
CD3D	1656	0.167563	0.481691
MAP3K7	1657	0.167553	0.489416
ITGB2	1709	0.165539	0.49454
IFNGR2	1752	0.163526	0.500014
IL18	1755	0.163455	0.507452
GZMA	1800	0.160945	0.512708
CD3G	1884	0.15733	0.515879
RPS9	2153	0.147134	0.509481
IFNAR2	2192	0.145526	0.514322
CD1D	2207	0.145031	0.52032
TLR2	2314	0.141137	0.521613
IL7	2322	0.140877	0.527764
LY75	2486	0.135906	0.526013
UBE2N	2490	0.135825	0.532128
SIT1	2547	0.133859	0.535545
HLA-DOB	2604	0.132209	0.538886

RIPK2	2643	0.13079	0.543047
CSF1	2645	0.130673	0.549022
GALNT1	2650	0.130565	0.554845
GZMB	2653	0.130495	0.560764
CD8A	2673	0.129907	0.565819
CD2	2705	0.128995	0.570241
STAT1	2778	0.126836	0.572548
IL11	2788	0.126444	0.577935
NCR1	2950	0.121294	0.575608
TLR6	3038	0.118896	0.576811
SPI1	3137	0.116205	0.577348
HLA-E	3242	0.113228	0.577453
IL12RB1	3251	0.112893	0.582265
NCF4	3368	0.109839	0.581623
CD40	3432	0.108251	0.583516
IL2	3520	0.106134	0.58413
IFNG	3641	0.103239	0.582987
HLA-DOA	3684	0.102199	0.585633
IL16	3686	0.102107	0.590292
CD3E	3689	0.102042	0.594898
CXCL9	3690	0.102041	0.599603
IRF8	3712	0.10152	0.603251
HLA-DMA	3731	0.101012	0.607023
EIF3D	3768	0.100127	0.609868
CD247	3931	0.096379	0.606344
STAT4	3936	0.096168	0.610581
GCNT1	3998	0.094731	0.611948
WARS	4010	0.094401	0.615759
RPL9	4066	0.092876	0.617336
EIF5A	4123	0.091467	0.618799
EGFR	4177	0.090371	0.620359
MMP9	4480	0.083956	0.609375
PRKCB	4496	0.083634	0.612494
CCL22	4537	0.082794	0.614343
FGR	4623	0.080898	0.613893
FASLG	4646	0.080372	0.616516
IL2RB	4658	0.080073	0.619667
TNF	4712	0.07874	0.62069

IL27RA	4822	0.07644	0.618853
HCLS1	4833	0.076246	0.621877
DYRK3	4914	0.074958	0.621398
IL6	4957	0.074388	0.622762

Abbreviations: ES, enrichment score

Supplementary Table 3. Core enrichment gene list of HALLMARK_INFLAMMATORY_RESPONSE

Probe	Rank in gene list	Rank metric score	Running es
GNAI3	14	0.35152	0.015873
MSR1	91	0.307499	0.026622
TNFAIP6	97	0.304343	0.040716
SLC31A2	140	0.292712	0.052441
C3AR1	151	0.28894	0.065562
CYBB	194	0.278465	0.076616
IFNAR1	202	0.277369	0.08934
CD14	222	0.27333	0.101283
AXL	231	0.271839	0.113698
AHR	233	0.271695	0.12645
FPR1	248	0.269305	0.138449
CCL7	280	0.263992	0.149362
CCL2	289	0.262089	0.161317
HRH1	291	0.261731	0.1736
IL10	297	0.260421	0.185624
RNF144B	445	0.240953	0.189744
CD48	537	0.231618	0.19618
ABI1	542	0.231367	0.206884
OSMR	585	0.2276	0.215541
LCP2	619	0.224889	0.224514
AQP9	641	0.223491	0.23401
ADORA2B	645	0.222933	0.244366
IL15	658	0.222156	0.254243
CMKLR1	663	0.221937	0.264503
IL1B	724	0.217414	0.271795
IL7R	740	0.216597	0.281262
INHBA	742	0.216479	0.291412
SLC31A1	773	0.214839	0.300059
CXCL10	832	0.211038	0.307148
NMI	902	0.205577	0.313439
TLR3	1039	0.19833	0.316093

LYN	1131	0.193173	0.320717
CALCRL	1142	0.192647	0.329302
CD69	1159	0.191855	0.337554
TNFRSF9	1164	0.191671	0.346388
C5AR1	1191	0.190695	0.354094
MEFV	1270	0.185784	0.35901
EMP3	1302	0.184054	0.366156
IL15RA	1397	0.17957	0.369992
GPR183	1425	0.178021	0.377052
PDE4B	1501	0.173767	0.381549
PTGER4	1576	0.170627	0.385947
IFNGR2	1752	0.163526	0.385042
IL18	1755	0.163455	0.392645
ITGB3	1904	0.156634	0.392744
CXCL8	1946	0.155144	0.398037
P2RX7	2166	0.146772	0.394177
IRAK2	2173	0.146297	0.400775
RHOG	2180	0.145934	0.407356
NAMPT	2232	0.143839	0.411624
IL10RA	2264	0.142639	0.416819
CXCL6	2270	0.142424	0.423284
IL1R1	2283	0.142002	0.429384
DCBLD2	2312	0.141169	0.434657
TLR2	2314	0.141137	0.441258
CXCL11	2396	0.138517	0.443799
PSEN1	2434	0.137378	0.448452
ITGA5	2470	0.136525	0.453162
ADGRE1	2479	0.136096	0.459181
SGMS2	2598	0.132394	0.459613
RIPK2	2643	0.13079	0.463611
CSF1	2645	0.130673	0.469718
ITGB8	2729	0.128329	0.471681
CXCR6	2753	0.127587	0.476561
MMP14	2804	0.125831	0.48003
NFKB1	2823	0.125139	0.48504
PDPN	2913	0.122258	0.486422
OLR1	3018	0.119514	0.486936
RGS1	3036	0.119027	0.491708

CD70	3056	0.118319	0.496348
CDKN1A	3225	0.113973	0.493452
TNFSF10	3263	0.11263	0.496939
APLNR	3306	0.111383	0.50012
CD40	3432	0.108251	0.499071
IL18R1	3441	0.10811	0.503771
RGS16	3477	0.10723	0.507101
SLAMF1	3573	0.104808	0.507366
OSM	3650	0.103158	0.508487
CXCL9	3690	0.102041	0.511376
MET	3696	0.101886	0.51593
SERPINE1	3771	0.100115	0.517007
KLF6	3816	0.099055	0.519509
SPHK1	3880	0.097503	0.521003
IL1A	3994	0.094797	0.51991
PLAUR	4075	0.092711	0.520343
MARCO	4114	0.091807	0.522799
LPAR1	4192	0.09006	0.523254
IRF1	4201	0.089817	0.527092
CCL24	4211	0.089575	0.53087
EIF2AK2	4244	0.089106	0.533493
PTGIR	4245	0.089103	0.537692

Abbreviations: ES, enrichment score

Supplementary Table 4. Core enrichment gene list of HALLMARK_IL6_JAK_STAT3_SIGNALING

Probe	Rank in gene list	Rank metric score	Running es
IFNGR1	17	0.346692	0.037449
FAS	65	0.315861	0.070024
IFNAR1	202	0.277369	0.093992
CD14	222	0.27333	0.123242
CD44	262	0.267245	0.150841
CCL7	280	0.263992	0.179158
ITGA4	394	0.246788	0.200876
CCR1	426	0.243185	0.22621
OSMR	585	0.2276	0.243605
IL10RB	613	0.225372	0.267168
IL1B	724	0.217414	0.285789
CXCL10	832	0.211038	0.303853
IL2RA	1241	0.187416	0.304571
IL15RA	1397	0.17957	0.31681
CSF2RA	1398	0.179544	0.336635
PTPN2	1481	0.174942	0.351937
MYD88	1631	0.168459	0.363243
TNFRSF12A	1694	0.166128	0.378551
IFNGR2	1752	0.163526	0.393816
IL13RA1	1839	0.159551	0.407223
ITGB3	1904	0.156634	0.421385
CD9	2012	0.15292	0.433031
IL1R1	2283	0.142002	0.435492
TLR2	2314	0.141137	0.449607
IL7	2322	0.140877	0.46482
CXCL11	2396	0.138517	0.47654
CBL	2611	0.131901	0.480627
CSF1	2645	0.130673	0.49344
BAK1	2688	0.129391	0.505671
STAT1	2778	0.126836	0.515318
HMOX1	2866	0.12371	0.524719

STAM2	3120	0.116709	0.525219
CD38	3197	0.114716	0.534164
HAX1	3212	0.114428	0.546114
IL12RB1	3251	0.112893	0.556719
IL18R1	3441	0.10811	0.559402
CXCL9	3690	0.102041	0.558528
CRLF2	3708	0.101586	0.568912
IL6ST	3730	0.101031	0.57904
CSF2	3930	0.096427	0.579944

Abbreviations: ES, enrichment score

Supplementary Table 5. Core enrichment gene list of HALLMARK_IL2_STAT5_SIGNALING

Probe	Rank in gene list	Rank metric score	Running es
IFNGR1	17	0.346692443	0.020213468
CD86	37	0.332375228	0.03945929
TWSG1	56	0.319727212	0.05798637
GSTO1	108	0.298998147	0.073631816
GPR65	118	0.296194255	0.09117273
AHR	233	0.271694928	0.10206189
CD44	262	0.267244816	0.11691064
IL10	297	0.260421157	0.13104999
LRRC8C	342	0.252861857	0.14423853
EMP1	536	0.231845245	0.1488227
CD48	537	0.231617928	0.16288549
FGL2	697	0.219984025	0.16842173
GLIPR2	796	0.213366598	0.17655638
CXCL10	832	0.211038142	0.18764824
CDC42SE2	889	0.2065956	0.19743751
PHTF2	965	0.202045798	0.20601603
TGM2	1135	0.193019524	0.20942329
TNFRSF9	1164	0.191671163	0.21968354
IL2RA	1241	0.18741639	0.22732466
SLC39A8	1304	0.184018791	0.23544808
CAPG	1414	0.17858769	0.2409301
TIAM1	1546	0.172013	0.2449309
ITGAE	1579	0.170540422	0.25371146
SPP1	1607	0.169419378	0.2626699
GBP4	1704	0.165596142	0.2680025
PLSCR1	1916	0.156069875	0.26710063
CDCP1	1957	0.154838249	0.27453437
SLC2A3	1971	0.154369667	0.2832676
IL10RA	2264	0.14263922	0.2775664
ICOS	2271	0.142409071	0.28591773
DENND5A	2293	0.141632333	0.29348415

NRP1	2456	0.136899829	0.29382834
P4HA1	2481	0.1360742	0.30090976
NFIL3	2561	0.133510321	0.3051304
CSF1	2645	0.130673066	0.30898204
NCS1	2737	0.128008068	0.3122784
NT5E	2952	0.121281981	0.3091168
PLIN2	3060	0.118163668	0.31102854
ARL4A	3113	0.116928421	0.31557035
HOPX	3187	0.11507012	0.31896648
BCL2L1	3220	0.114106551	0.32432064
IKZF2	3239	0.113301486	0.3303145
TNFSF10	3263	0.112630233	0.33602166
ITGA6	3359	0.110071741	0.33803228
ALCAM	3422	0.108552597	0.34157372
IL18R1	3441	0.108110197	0.34725237
RGS16	3477	0.107229769	0.35204145
AHCY	3612	0.103982903	0.35176423
IRF8	3712	0.101520345	0.3530589
CASP3	3772	0.09996976	0.35622677
KLF6	3816	0.099054717	0.36012605
CSF2	3930	0.096426584	0.36042288
CYFIP1	4088	0.092226788	0.35830066
PDCD2L	4097	0.092071116	0.36349732
CST7	4213	0.089547895	0.36327815
SNX14	4237	0.089212097	0.3675635
PNP	4285	0.088312574	0.3706138
HK2	4291	0.088128977	0.37571868
BATF3	4320	0.087463118	0.3796519
WLS	4325	0.087369435	0.38475984
SERPINB6	4563	0.08223594	0.3780963
CD83	4633	0.08066383	0.3796002
IL2RB	4658	0.080072574	0.38328144
SWAP70	4734	0.078246631	0.38434345
CTSZ	4761	0.077607751	0.38777667
SMPDL3A	4983	0.073874943	0.38139245
MXD1	5139	0.070630133	0.37805736
COL6A1	5182	0.070068054	0.38024586
TNFRSF1B	5284	0.068089865	0.3794124

F2RL2	5301	0.067844041	0.38274467
TTC39B	5327	0.067401282	0.38560736
EOMES	5472	0.064839274	0.3824617
TNFRSF8	5481	0.064715587	0.38599744
IL1RL1	5545	0.063398793	0.38674816
PIM1	5736	0.060215853	0.38105935
CD79B	5749	0.059957873	0.3841095
ENPP1	5783	0.05940235	0.38609308
ADAM19	5878	0.057772603	0.38497752
SELP	5880	0.057671446	0.38842988

Abbreviations: ES, enrichment score

Supplementary Table 6. Gene sets within the top 20-ranked list related to low HIP1R in squamous cell carcinoma patients

NAME	SIZE	ES	NES	Nominal p-val	FDR q-val	FWER p-val
HALLMARK_MTORC1_SIGNALING	165	0.627194	2.298721	0	0	0
HALLMARK_PROTEIN_SECRETION	75	0.595903	2.115246	0.001912	0.004501	0.005
HALLMARK_FATTY_ACID_METABOLISM	136	0.473653	1.822678	0.005859	0.088537	0.117
HALLMARK_ANDROGEN_RESPONSE	71	0.455162	1.81704	0.003899	0.067781	0.119
HALLMARK_GLYCOLYSIS	164	0.434955	1.774345	0.00789	0.083118	0.162
HALLMARK_ADIPOGENESIS	158	0.43747	1.768937	0.007752	0.071546	0.167
HALLMARK_MYC_TARGETS_V1	163	0.58844	1.76365	0.027451	0.064392	0.173
HALLMARK_REACTIVE_OXYGEN_SPECIES_PATHWAY	39	0.561454	1.756679	0.026749	0.058249	0.179
HALLMARK_OXIDATIVE_PHOSPHORYLATION	148	0.585247	1.749335	0.023576	0.054867	0.186
HALLMARK_HEME_METABOLISM	153	0.357383	1.648846	0.003831	0.100822	0.329
HALLMARK_UNFOLDED_PROTEIN_RESPONSE	87	0.426965	1.635483	0.017274	0.097984	0.347
HALLMARK_ALLOGRAFT_REJECTION	167	0.512914	1.602405	0.053608	0.111173	0.404
HALLMARK_BILE_ACID_METABOLISM	89	0.438328	1.597392	0.015656	0.105251	0.411
HALLMARK_UV_RESPONSE_UP	131	0.34247	1.519016	0.010121	0.155365	0.551
HALLMARK_INTERFERON_GAMMA_RESPONSE	166	0.511589	1.484784	0.098326	0.173868	0.6
HALLMARK_PEROXISOME	88	0.359202	1.44817	0.040307	0.19454	0.656
HALLMARK_COMPLEMENT	170	0.408295	1.440125	0.109344	0.190602	0.676
HALLMARK_CHOLESTEROL_HOMEOSTASIS	62	0.390861	1.439454	0.077535	0.180709	0.677

HALLMARK_EPITHELIAL_MESENCHYMAL_TRANSITION	160	0.46266	1.416673	0.16701	0.191798	0.714
HALLMARK_XENOBIOTIC_METABOLISM	170	0.346733	1.39775	0.06383	0.199909	0.737

Abbreviations: ES, enrichment score; FDR, false discovery rate; family-wise error rate, FWER; NES, normalized enrichment score

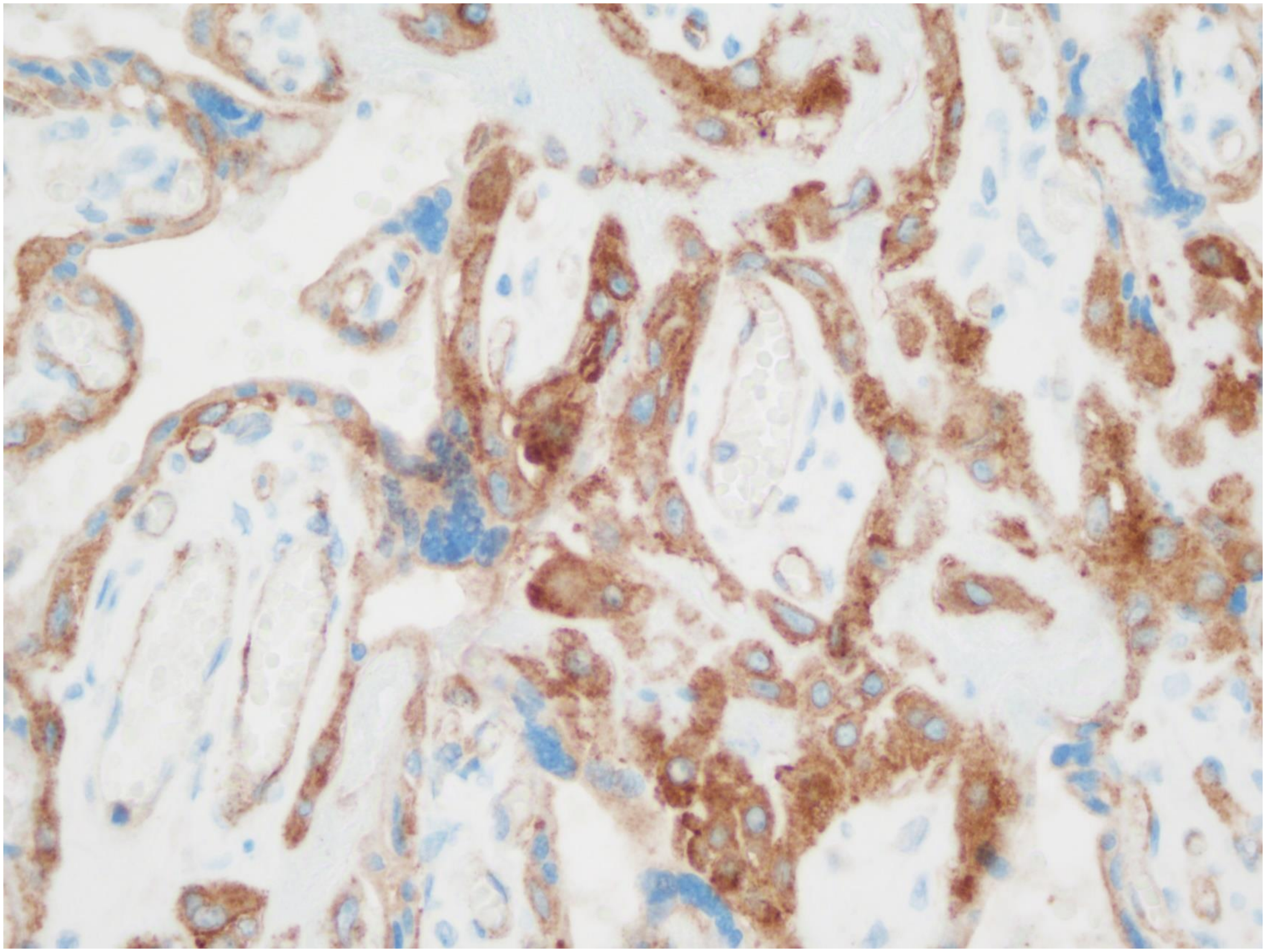

Figure S1. HIP1R expression in positive control. Positive HIP1R expression in placental tissue, X400.

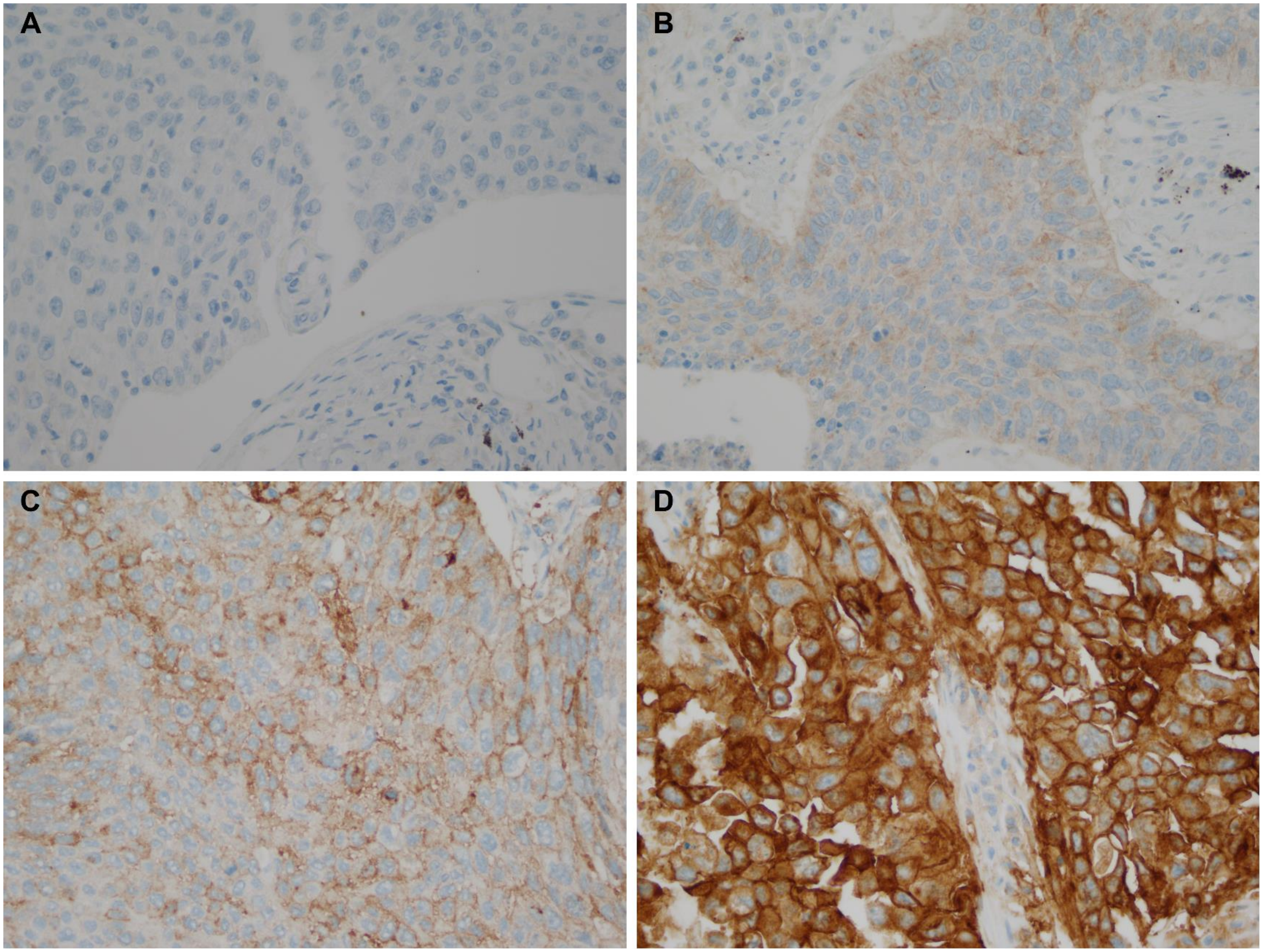

Figure S2. PD-L1 sp263 expression in non-small cell carcinoma. (A) No staining of PD-L1 sp263, x400. (B) Faint PD-L1 sp263 staining, x400. (C) Moderate PD-L1 sp263 staining, x400. (D) Strong PD-L1 sp263 staining, x400.

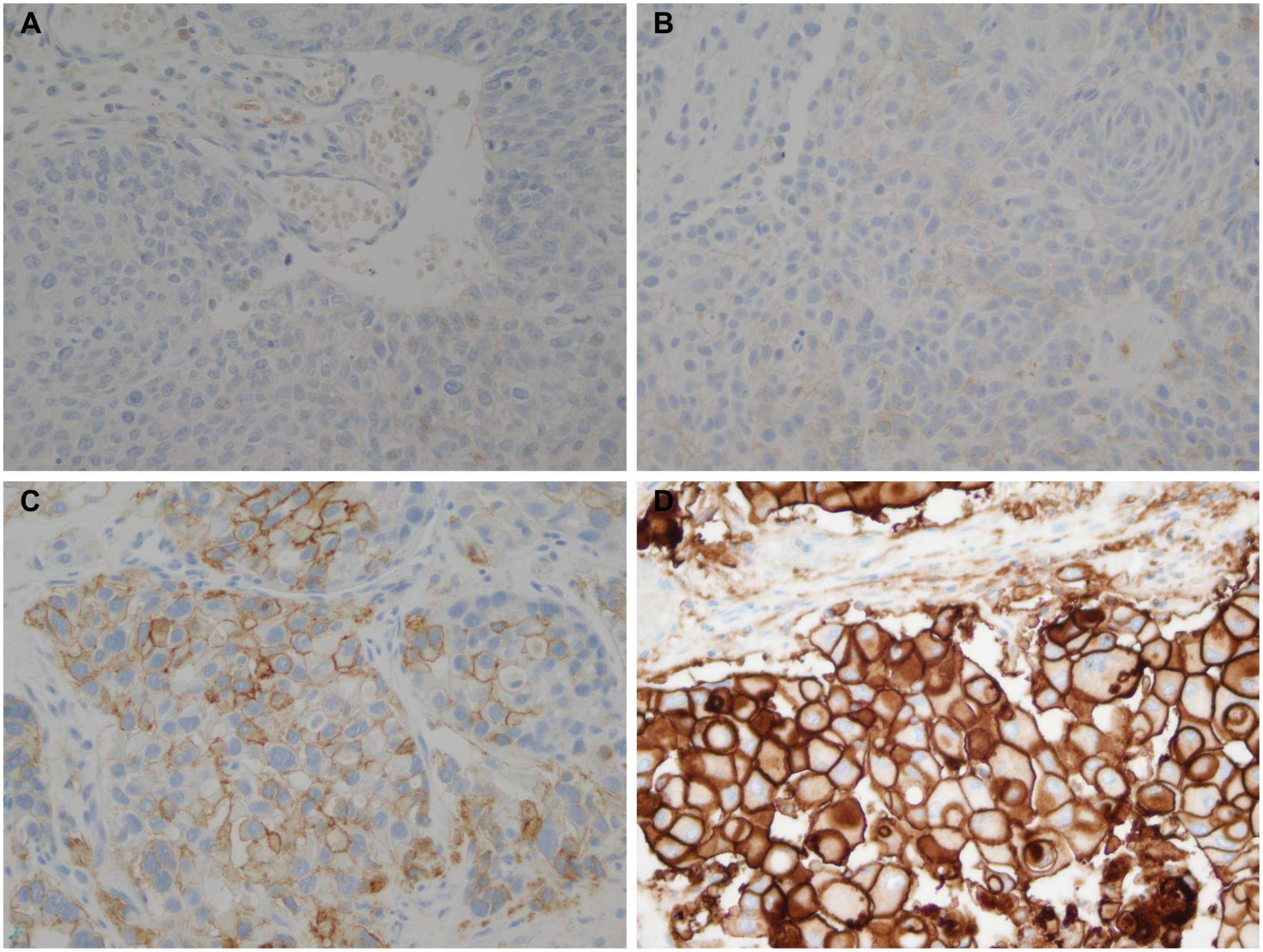

Figure S3. PD-L1 22c3 expression in non-small cell carcinoma. (A) No staining of PD-L1 22c3, x400. (B) Faint PD-L1 22c3 staining, x400. (C) Moderate PD-L1 22c3 staining, x400. (D) Strong PD-L1 22c3 staining, x400.

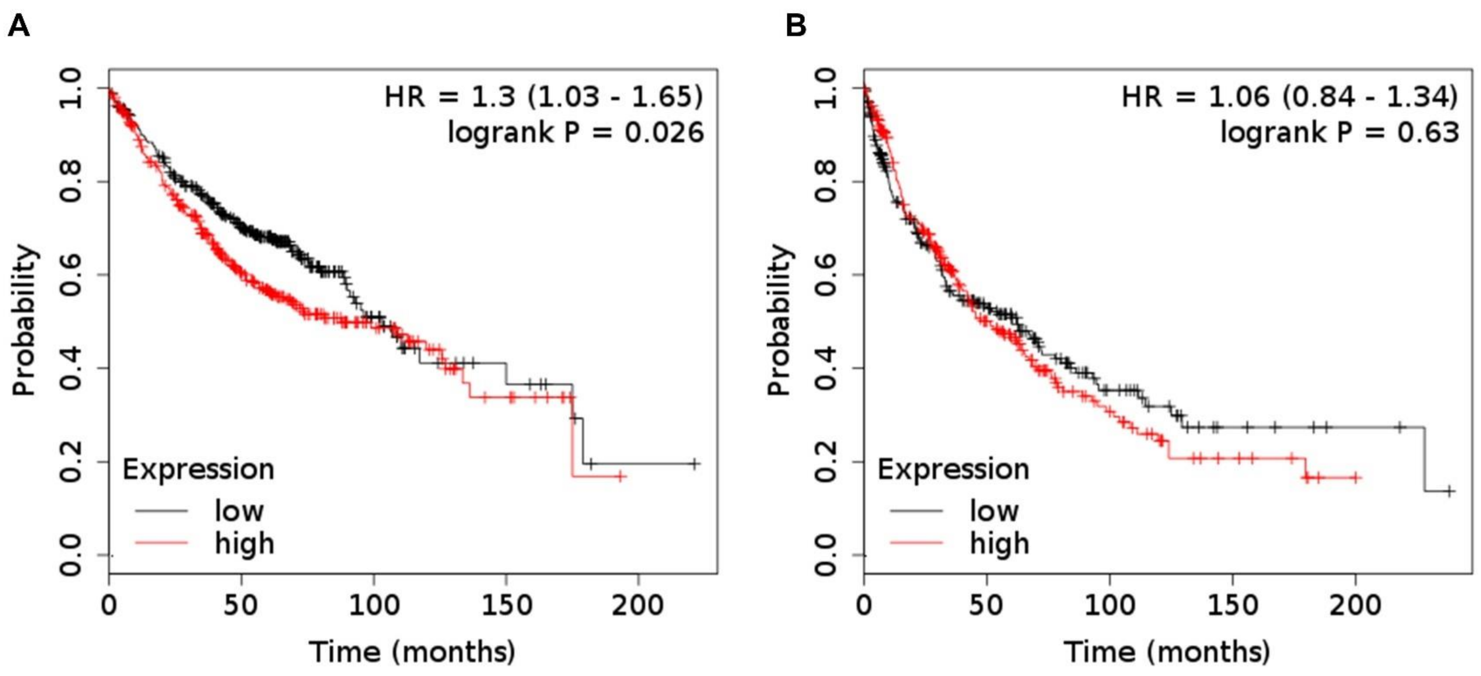

Figure S4. Comparison of survival rates, according to *HIP1R* mRNA expression in patients with non-small cell carcinoma. (A) Overall survival (OS) and HIP1R in lung adenocarcinoma. (B) OS and HIP1R in lung squamous cell carcinoma.
